# Social representations of mask wearing in the general population during the COVID-19 pandemic

**DOI:** 10.3389/fpubh.2023.1136980

**Published:** 2023-04-24

**Authors:** Elvire Bornand, Frédérique Letourneux, Colin Deschanvres, David Boutoille, Jean-Christophe Lucet, Didier Lepelletier, Brice Leclere, Séverine Mayol, Nathan Peiffer-Smadja, Gabriel Birgand

**Affiliations:** ^1^Centre Nantais de Sociologie (CENS), Université de Nantes, Nantes, France; ^2^Centre G. Simmel, Ecole de Hautes Etudes en Sciences Sociales, Rennes, France; ^3^Department of Infectious Diseases, University Hospital of Nantes and CIC 1413, INSERM, Nantes, France; ^4^Equipe de Prévention du Risque Infectieux, Claude Bernard Hospital, Assistance Publique—Hôpitaux de Paris, Paris, France; ^5^Unité de Gestion du Risque Infectieux, Centre Hospitalo-Universitaire de Nantes, Nantes, France; ^6^Department of Medical Evaluation and Epidemiology, CHU Nantes, Nantes, France; ^7^Université Paris Cité and Université Sorbonne Paris Nord, Inserm, IAME, Paris, France; ^8^Infectious and Tropical Diseases Department, Bichat—Claude Bernard Hospital, Assistance Publique—Hôpitaux de Paris, Paris, France; ^9^NIHR Health Protection Research Unit in Healthcare Associated Infection and Antimicrobial Resistance at Imperial College London, Hammersmith Campus, London, United Kingdom; ^10^Center for the Prevention of Healthcare Associated Infections Pays de la Loire, Nantes, France

**Keywords:** COVID-19, mask, facemask behavior, infection control, general population, qualitative study analysis, sociology

## Abstract

**Introduction:**

Although one of the most prominent interventions against COVID-19, face masks seem poorly adopted by the general population. A growing body of literature has found that using face masks has social meaning. This qualitative study assessed the perceptions, representations and practices of mask wearing in the general population.

**Methods:**

A qualitative survey by short semi-structured walking interviews was carried out from April to December 2021 in 11 cities in France's Pays de la Loire region. Study locations were selected for their varied geographical, social, and economic characteristics, with urbanized and rural areas. Four domains linked to perceptions of masks and wearing them were explored: (i) evolution in mask wearing, (ii) decision-making methods for wearing and not wearing; (iii) incorporating the mask into way of life; (iv) projecting into the future.

**Results:**

A total of 116 people were interviewed. Masks marked a shift from the ordinary world to the pandemic. Overall, interviewees considered masks an obstacle to breathing, communication, and social interactions, leading to establishing strategies circumventing the mask mandate. Poor attention was paid to their medical usefulness as an obligatory clothing accessory. Mask-wearing decisions were driven by social relations, common sense, and vulnerability. The greater the feeling of security (i.e., being with close relatives), the less it was worn or worn properly, with decreased attention to others and their health. Most participants did not remember learning to wear a mask. Some were convinced that mask-wearing could not be learned (experiential knowledge). Institutions (school and work) played a central role by facilitating incorporation of masks into daily life.

**Conclusions:**

This study emphasizes the need to reinforce the individual medical values of face masks to prevent COVID-19. Ambitious education and training programmes should be planned to learn how and when to wear masks. Institutions (work and school) may be critical for this purpose.

## Introduction

Face masks are one of the most prominent interventions against COVID-19 (1). In many countries, masks became mandatory in crowded areas where social distancing could not be maintained (such as transport) and were recommended outdoors (2). According to a recent systematic review of the literature, wearing a mask is associated with an almost 50% reduction in the incidence of COVID-19 (3). Furthermore, the mean mask-wearing levels observed in a large survey from regions across six continents has made it possible to estimate a 19% decrease in the reproduction number (4). The widespread use of surgical masks is therefore essential for protecting the population.

Before the COVID-19 pandemic, the French population was not used to wearing masks, even in winter during influenza season. Masks were limited to healthcare professionals, especially in a hospital setting, when caring for patients with transmissible diseases (5, 6). Following national and local mandatory measures to control the spread of COVID-19, the general population had to adopt a set of provisions (distancing) and devices (masks), in a very short time. A national regular survey described increased adoption of systematic mask wearing in the general population from 15% in March to 85% in November 2020, triggered by mandates (7). Trends following this period showed a significant decrease to 66–73% during winter 2021 and to 34% in May 2022. In a previous study gathering 3,354 observations from June to July 2020 in western France, 56% of individuals were wearing a mask in public spaces. The factors independently associated with wearing a facemask were being indoors, being in a mandatory area, female gender, age 41–65 years and >65 years (8).

The emergence of new variants or the occurrence of new epidemic waves have raised questions about protective measures against COVID-19 (9). Moreover, supply difficulties, changes in government decisions, the introduction of vaccination and general fatigue for complying with recommended barriers in the population may cause deviations in practices during epidemic phases. Few studies have been conducted on the sociological patterns of populations in terms of mask wearing and none have been conducted in a pandemic context. A Cochrane review of the barriers and facilitators of compliance with recommendations for the prevention of respiratory infections concluded that methodologically rigorous mixed method studies are needed, in the context of COVID-19, in hospitals but also in nursing homes and the community (10). The current way of exploring and explaining behaviors around mask use focuses on a normative perspective that seeks to highlight deviations from the rule. However, a comprehensive approach may better consider the practices and mechanisms of appropriation of masks by people themselves.

In this qualitative sociological study performed in the general population in diverse contexts in France, we investigated (i) the representations and perceptions of masks, (ii) the incorporation of masks into daily life, and (iii) how the usefulness of good compliance with mask-wearing rules with regard to controlling the pandemic is justified (or not).

## Methods

### Study design and setting

**Figure 1:**
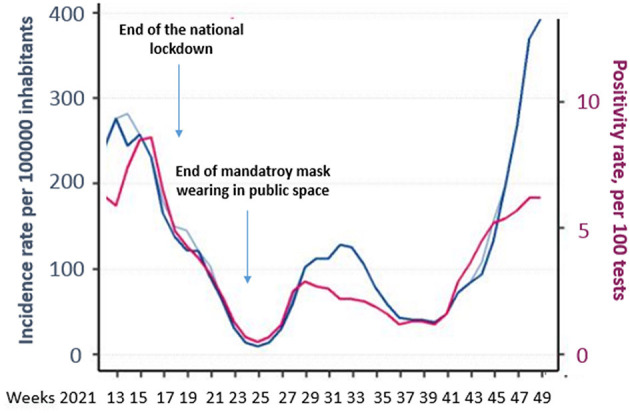
Incidence of COVID-19 in the Pays de la Loire region throughout the study period.

A qualitative study using walking interviews was carried out in outdoor public areas from April to December 2021 in 11 cities in the Pays de la Loire region in Western France, with a population of 3.8 million individuals (Supplementary Figure 1). The study period was characterized by the end of the lockdown period on 3 May 2021, and the end of mandatory masks in outdoor public spaces on 17 June 2021 (Figure 1). The interviews were done during various time periods in the day (morning and afternoon) and in various areas spread across the region: rural and urban (cities with >10,000 and with <10,000 inhabitants). Individuals present in the public area during the study periods were approached at random by two sociologists (EB and FL) and asked if they were willing to participate. The random and open process of selecting this convenient sample in outdoor spaces has the advantage of reaching diverse profiles. The sampling method considered gender and age, the only two variables that can be observed first hand to obtain a variety of profiles. The choice of a random sample in a public area was justified by the social facts observed. Thus, wearing a mask was discussed in a context where mask wearing was mandated (the public space) and where the person interviewed was not likely to have changed their behavior for the sake of social conformity in their relationship with the interviewers. The short semi-structured walking interviews made it possible to include a large number of participants and provided opportunities for collecting the spontaneous expression of representations and practices in the specific context approached (11).

### Data collection

The interview guide was designed to approach, through experience, the representations and practices related to mask wearing (Table 1). We sought to observe the potential existence of routinized practices, representations, and habits that were part of the appropriation of mask wearing by the general population. More specifically, the areas explored during the interview were: (i) evolution in representations and perceptions, (ii) the decision-making methods for wearing and not wearing a mask, (iii) practices and the degree of incorporation of masks into daily life, and (iv) the projection toward the future regarding mask wearing. The interview guide was first tested and validated on a panel of individuals, and then applied to all participants. Spontaneous probes were used, and additional questions were asked by researchers on a case-by-case basis depending on the statements made by the interviewee or to clarify the meaning of a participant's answers. The data were collected in public spaces in cities selected during the quantitative survey phase. Two senior sociologists (EB, FL) collected the data. Recruitment was allowed to continue until thematic saturation was achieved, i.e., until no new major themes were identified in the data (12). Responses were recorded using a digital recorder. The device was completed by live notetaking, with particular focus on non-verbal language. One interviewer took notes while the other asked questions and recorded the interview. Each interview was audio-recorded anonymously in addition to the notes taken by the researchers.

**Table 1:** Interview guide.

**Questions**	**Objectives**
Do you remember the first time you put on a face mask? Tell me	Assess how wearing a mask is rooted in the imagination and how it is narrated by the person interviewed. Collect details about state of mind.
Under what circumstances do you wear the mask? How do you decide whether or not to wear the mask? Indoors and outdoors.	Identify the main uses and meaning that the interviewee gives.
If we take the example of today, can you tell me in a little more detail how it happened? Starting with where you store the mask and when you put it on.	Anchor the uses of wearing a mask in a concrete example to be collected in as much detail as possible.
In your opinion, are you wearing your mask properly? How did you learn to wear it? How long do you wear it? Do you wear it differently today than last summer?	Clarify the evaluation criteria and the importance granted or not to the correct wearing of the mask. Also see whether or not there is any slacking off in mask-wearing and how it is justified.
Imagine that we are chatting and the tie of your mask breaks, what do you do?	Indirectly, complete the information collected on uses by seeing if the person has planned anything to deal with incidents (emergency mask for example).
How long do you think we're going to wear masks?	Using a more open approach, collect elements on the state of mind of the person—to consolidate the elements collected previously.

### Data analysis

Data were analyzed using an inductive thematic approach to openly explore the perceptions, representations, and practices regarding mask wearing in the general population (13). Audio recordings of each interview were listened to independently twice by two researchers (EB, FL), and information was triangulated with notes. All the interviews were described, and only the salient elements of each interview were transcribed. A thematic analysis was conducted in four stages. First, the responses obtained for each question were described and coded. After interviews had been coded, the researchers met and compared their coding, discussing each code and theme in detail and generating a final consensual code. This involved revisiting the raw data to confirm shared and consistent understanding of how the codes and themes were being used. Recurring themes were identified and categorized. The original data were then used to explore certain themes of interest in greater depth. Finally, the verbatim statements that best illustrated the analysis were chosen. This selection of verbatim was made not only taking the illustrative qualities of the quote into account in terms of content, but also the speaking skills of the respondents.

### Ethics statement

No ethics or Data Protection Committee Approval was required in France for this qualitative research as it did not come within the scope of application of the French Public Health Code.

## Results

A total of 116 participants were interviewed. The saturation effect was reached halfway through the interview campaign. Of the participants, 70 (60%) were women, with a mean age of 51 years (min-max: 14–90), 47 (40%) were retired and 38 (32%) were living alone (Table 2). The interviews lasted a median of 12 min, varying from 6 to 20 min. Mask wearing was related to four key themes: (i) a marker of a shift from the ordinary world to the pandemic, (ii) a daily burden more than a medical solution, (iii) a decision-making process driven by social relations, common sense, and vulnerability, and (iv) institutions as a lever of incorporation into daily life.

**Table 2:** Demographic characteristics of the participants.

**Characteristics**	***N* (%)**
**Age categories (years)**	
< 20	7 (6)
20–29	16 (14)
30–39	17 (15)
40–49	14 (12)
50–59	14 (12)
60–69	22 (19)
70–79	19 (16)
≥80	7 (6)
**Home life**	
Single	38 (33)
Couple	40 (34)
Couple with children	29 (25)
Adult alone with children	9 (8)
**Activity**	
In training	12 (10)
Working person	55 (47)
Inactive	2 (2)
Retired	47 (41)

### Face masks as a marker of a shift from the ordinary world to the pandemic

During the interviews, masks were one of the main markers of the pandemic, referring to the health situation as a whole. In the narrative of the pandemic, wearing a mask was associated with the feeling of shifting from the ordinary world to the pandemic world (Table 3, Q1-2).

“*Well, it's a total change, I wasn't expecting it at all. It was in March last year, 14 months ago*.”—Man, single, age 39, cleaner.

**Table 3:** Themes and illustrative data.

**Themes**	**Sub-themes**	**Illustrative quotations**
Face masks as a marker of a shift from the ordinary world to the pandemic.	Marker of a shift	Q1: *Well, it's a total change, I wasn't expecting it at all. It was in March last year, 14 months ago*.—Man, single, age 39, cleaner.
		Q2: *No… [Don't you remember?] it was in March, April, March that we started. Because wait… we didn't use masks straight away… I don't know!—*Woman, in a relationship, age 64, retired.
	Activities as a memory activator for the first time wearing a mask	Q3: *It was from when it first became obligatory, but to be honest, I don't remember when that was, it didn't stand out. I didn't see it as a constraint. The first time I wore one was to go to work because I wasn't in lockdown for work so I was working then. But I don't remember the first time I did it or the date*—Man, in a relationship, with children, age 37, member of the armed forces.
		Q4: *For me, I think it was for going to uni, before it was shut down. Just after the first lockdown… or no, it must have been during the summer season. Because unis were shut during the first lockdown and then there was the summer and masks were recommended, in certain places and obligatory. That was the first time I wore one*—Woman, single, age 22, sales assistant.
	Difficulties in obtaining masks	Q5: *It was right at the start of the lockdown, cloth masks that we sewed ourselves, in a family workshop. We care about the environment and immediately got out our bits of material and sewed masks together*—Man, married with children, age 53, photographer.
		Q6: *I don't remember any more, it was during the first lockdown, I don't remember exactly. I know it was hard to find them, though. At one point, I got one from the chemist's and I made it last for several days*—Woman, single, age 77, retired.
		Q7: *When did we go into lockdown the first time? In March 2020… then it must have been in May 2020… I have a neighbor who very kindly made me a cloth mask… You couldn't find them anywhere… It was a really hard thing to find—*Woman, single, age 61, retired.
		Q8: *I made my first one myself from a rubbish bag and a wipe. I'm 76 years old and I have diabetes, so I was at risk of dying. I live in Corsica, so at that time, there was a cluster, some patients even had to be evacuated by the Navy, it was really stressful*—Man, in a relationship, age 76, retired, Corsica.
	Emotions felt when wearing the mask	Q9: *Heavens! Absolutely not, I really don't remember [the first time I wore a mask]! It's really not nice having something covering your mouth and nose…—*Woman, single, age 77, retired.
A daily burden more than a sanitary solution.	An obligatory clothing accessory	Q10: *It's not so bad in winter as it keeps you warm, but when it's hot, it's unpleasant to wear—*Woman, single, age 77, retired.
	Obstacle to breathing, to communication, and a social barrier	Q11: *It's really not nice when your mouth, nose, whatever are covered up—*Woman, single, age 77, retired.
		Q12: *I wear it on my hand. I decided I wasn't going to wear it because I can't breathe properly with it…*—Man, non-mask-wearing, in a relationship, age 50, artist.
		Q13: *When I go out for a walk, I wear it under my chin and put it on properly when I pass someone […] When I'm outside, it's still more comfortable to not wear one*—Woman, in a relationship, age 61, retired.
	Rhetoric of freedom	Q14: *It infringes on my personal freedom, first and foremost because I can't breathe freely*—Woman, in a relationship, age 66, retired.
		Q15: …* I call it a muzzle. There's a woman who said the next step would be a leash -*Woman, non-mask wearer, in a relationship, age 66, retired.
	Poor attention paid to the medical usefulness	Q16: *I wear it for an hour or a half-hour to go and get my newspaper or the bread*—[Wears the same mask since the start of the obligation to do so]—Man, single, age 83, retired.
		Q17: *I wear the same one several days in a row… because if I only use it for an hour or two, it can still be used the next day—*Woman, single, age 72, retired.
	Strategies for circumventing the mask mandate	Q18: *If you want to take your mask off, you go and sit at the terrasse of a café—*Woman, mask-wearing, in a relationship, with children, age 41, homemaker.
		Q19: *I smoke, I drink, I take it off…—*Man, non-mask-wearing, single, age 23, student.
		Q20: *Now, when we see each other in our group of friends, we're all vaccinated or immunized*—Woman, single, age 73, retired.
	Conspiratorial opinions	Q21: *How are we supposed to know? Some say you need to wear them, others that you absolutely don't… and we don't understand anything anymore. Some of them are positive, some of them negative. It's always like that with them. […] But as far as I'm concerned, they always do what they want. I couldn't care less—*Man, single, age 62, unemployed.
		Q22: *We're not out of the woods yet. Most people don't question any of these decisions, they just keep on submitting to them… even if I'm not sure even they're convinced of the efficacy. We're heading straight into a sort of health dictatorship that is increasingly strict and irrational. But I don't trust our government anymore, in the society we're living in. Now, I'm more in a vision of*
		*spending time with people who share the same values as me more or less and we're trying to create a parallel society, to get out of this crazy system—*Woman, in a relationship, with children, age 41, office worker.
	Desire to “regain a lost freedom”	Q23: *I think we've got a few more years of this to go, I don't think that as soon as the pandemic's over we'll be told to stop wearing masks. I think masks will become part of everyday life*—Woman, in a relationship, with a child, age 39, IT project manager.
		Q24: *It's going to be become a habit whenever we get sick, like in Asian countries, it's here to stay—*Woman, mask-wearing, single, age 25, teacher.
A decision-making process for mask wearing driven by social relations, common sense, and vulnerability.	Exposure to the known and unknown	Q25: *I wear a mask all the time, it's systematic, when I'm in the street, in shops… Even in the hall of my building when I get home—*Woman, age 24, postal worker.
		Q26: *I don't wear one when I'm outside, but in the center, I do, when there are loads of people and we can't necessarily avoid them—*Man, in a relationship, with children, age 40.
	Behaviors in the private sphere	Q27: *When we go to friends' houses, we don't wear masks but at the same time, we don't get too close to each other and we don't hug—*Man, in a relationship, age 64, retired.
		Q28*: With my friends and family I don't wear a mask, I only wear it when it's obligatory, outside or in shops… but between you and me, we're careful, we don't kiss or anything like that, but we don't wear masks—*Woman, age 22, market stall seller.
		Q29: *I don't wear one when I have visitors. We tend to believe that the risks are lower, plus we also know that we'll end up having drinks together and will have to take it off then anyway—*Woman, mask-wearer, single, age 23, doing civic service.
	“Common sense”	Q30: *I'm not a scientist, I trained as a pastry chef originally, but I've studied how bacteria proliferate and I think that wearing masks is more like a culture medium than a barrier. You just need to think a little bit logically to know that it's all crap. Maybe I'm wrong and maybe it is a really serious epidemic and everything… But I go out every day and I see the same people every day and, well, I'm sorry to say this, but no one has disappeared in a year—*Man, single, age 32, unemployed.
	Vulnerability	Q31: *We wore masks at Christmas with my whole family, it was really weird… sitting around the table at my grandparents' house. We'd never done it before, it was really strange, it felt clinical when we talked with each other. And it felt weird doing it with close family at a family dinner. But with grandparents and anyone a little bit vulnerable, obviously we tried to be careful… Otherwise, with friends, no, we didn't wear masks*—Woman, single, age 26, student.
		Q32: *I wear the same one several days in a row… because if I only use it for an hour or two, it can still be used the next day—*Woman, single, age 72, retired.
	Generational boundaries	Q33*: When I go and see my grandparents, I also get myself tested—*Woman, lives with her family, age 17, high school student.
		Q34: *In Lyon, we wanted to go and see our grandchildren, but my son said it would be better for us to be vaccinated before going, so we delayed our trip. We didn't even kiss our grandchildren (even though they'd been tested). We used protective measures—*Woman, in a relationship, age 65, retired.
		Q35: *For parties or birthdays, we got tested. For Juliette's 18th birthday, last time, the test was obligatory, PCR or antigen […] because it was when there were people from other high schools […]. We got tested 2 days before the party and took care to not see other people in between. And then, during the party, we didn't wear masks anymore. We also tried to not mix glasses and plates, we wrote our names on them—*Woman, living with her family, age 17, high school student.
Role of institutions in facilitating the incorporation of the mask into daily life.	Dissemination of knowledge and good behaviors	Q36: *At work, we had rubbish bins for biological waste: masks and wipes. We had a protocol when we took a case or returned it—*Woman, single with children, age 41, salesperson.
		Q37: *In class, we didn't have a choice, when we're eating, we take them off and when it's really hot, I wear it under my nose—*Woman, lives with her parents, age 17, high school student.
		Q38: *When we went back to school, they taught us how to wear our masks properly […] while before we wore them any old way. We had one for mornings and one for afternoons, and we had to change them at lunchtime—*Man, living with his family, age 15, high school student.
	Learning to wear a mask	Q39: *Learn how to wear them, no? But after that, I have no idea if I wore them correctly or not—*Woman, in a relationship, age 45, farmer.
		Q40: *Honestly, I just put it over my face, the way I think feels right, covering my nose and mouth properly*—Woman, single, age 24, postal worker.

**Figure 2:**
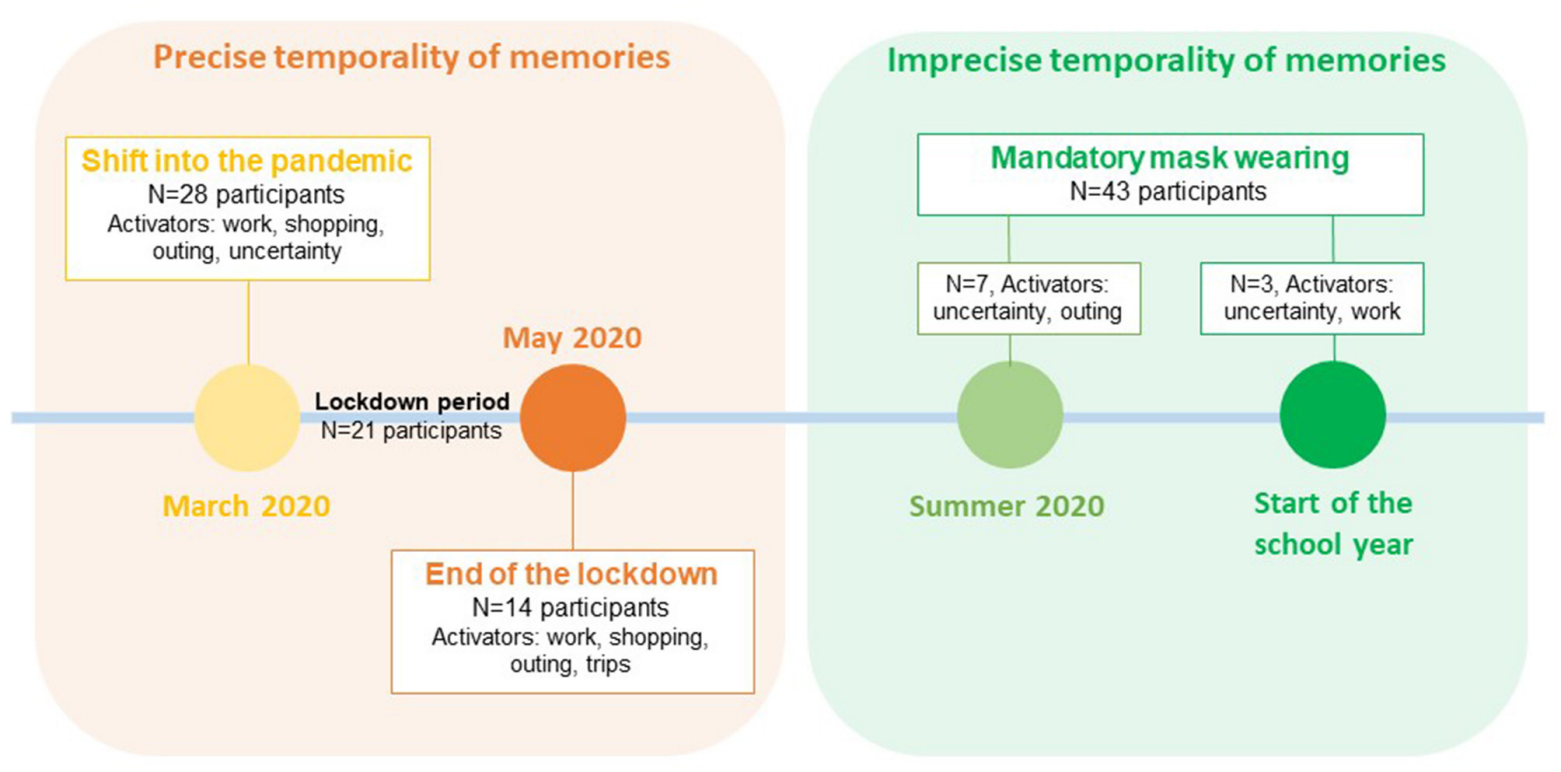
Sensitivity chronology displaying memory activators according to the period of first masks wearing. The number in each black rectangle indicates the number of interviewees who shared this time representation.

Participants were not aligned on the date or period which could in itself qualify the shift into the pandemic situation. Figure 2 shows the number of participants in relation to the memory of the date masks were first worn. Of the 116 participants, 63 (54%) situated the first experience of mask wearing in a period between March and May 2020 corresponding to the first lockdown in France. Memories associated with this first period were often precise and situated chronologically. The remaining participants (*n* = 53, 46%) situated “their first time” from summer 2020 to the start of the school year in September 2020. Memories were less precise and were marked by the notion of obligation.

Three phenomena seemed to play the role of memory activator for the first time masks were worn: the activity carried out while wearing the mask, the availability of masks and supply, and the feeling when wearing a mask (Table 3, Q3–9). The first activator related to agency and activities while wearing a mask, and at the end of the first lockdown, to work conditions, or shopping, with a rhetoric of change.

“*For me, I think it was for going to uni, before it was shut down. Just after the first lockdown… or no, it must have been during the summer season. Because unis were shut during the first lockdown and then there was the summer and masks were recommended, in the Port of Pornic for example. That was the first time I wore one*”—Woman, single, age 22, sales assistant.

The second memory activators related to the difficulty in obtaining masks in spring 2021. Mutual aid within families and in neighborhoods was used to make cloth masks. Patterns or tutorials for making reusable masks with the least impact on the environment were all positive points mentioned.

“*It was right at the start of the lockdown, cloth masks that we sewed ourselves, in a family workshop. We care about the environment and immediately got out our bits of material and sewed masks together*”—Man, married with children, age 53, photographer.

The last memory activator was linked to the emotions felt when wearing the mask, mainly discomfort, the intensity of which varied from one person to another, going as far as feelings of suffocation.

“*Heavens! Absolutely not, I really don't remember [the first time I wore a mask]! It's really not nice having something covering your mouth and nose…*”—Woman, single, age 77, retired.

### A daily burden more than a medical solution

The medical reason for mask wearing was mentioned less than the feeling of constraint (Table 3, Q10–24). For all participants, the emotions associated with wearing a mask were quite negative. The perception of the mask as an obligatory clothing accessory rather than a piece of protective equipment was widely shared within the population interviewed. This also resulted in a mask worn under the nose or under the chin.

“*It's not so bad in winter as it keeps you warm, but when it's hot, it's unpleasant to wear*”—Woman, single, age 77, retired.

The mask was presented as an obstacle to breathing and communication, and a social barrier overall.

“*It's really not nice when your mouth, nose, whatever are covered up*”—Woman, single, age 77, retired.

The rhetoric of freedom was used to denounce mask-wearing.

“…* I call it a muzzle. There's a woman who said the next step would be a leash*”—Woman, non-mask wearer, in a relationship, age 66, retired.

The poor attention paid to the medical usefulness of masks was characterized by participants' practices. Some stated that they wear a single-use mask repeatedly for several days, others said they put their mask in their pocket once removed, most often simply rolled into a ball, or stored in their bag.

“*I wear the same one several days in a row… because if I only use it for an hour or two, it can still be used the next day*”—Woman, single, age 72, retired.

Drinking, eating or smoking were used as strategies for circumventing the mask mandate. These pretexts were sometimes sources of tension between those who practice them and others.

“*If you want to take your mask off, you go and sit at the terrasse of a café*”—Woman, mask-wearing, in a relationship, with children, age 41, homemaker.

Young participants used different techniques to maintain their social activities without wearing a face mask while still trying to protect themselves and others from the epidemic. They restricted their social circle, by meeting up in small groups formed only of students from the same school. In April and May 2021, SARS-CoV-2 testing was mentioned as a solution for withdrawing the mask during festive events. From June 2021, the vaccine replaced the test as a barrier to transmission. For large events, a quarantine was applied in previous days to prevent “last minute” contaminations.

“*Now, when we see each other in our group of friends, we're all vaccinated or immunized*”—Woman, single, age 73, retired.

Some conspiratorial opinions were recorded, but remained an extremely small minority (*n* = 7, 6%). Most of the protest speeches targeted governmental measures and the confusion generated around the credit to be given to health measures.

“*How are we supposed to know? Some say you need to wear them, others that you absolutely don't… and we don't understand anything anymore. Some of them are positive, some of them negative. It's always like that with them. […] But as far as I'm concerned, they always do what they want. I couldn't care less*”—Man, single, age 62, unemployed.

For most participants, the desire to have the mask-wearing obligation lifted was expressed with a certain impatience, and a desire to “regain lost freedom.” However, all the interviews revealed a rather pessimistic vision of the future, one in which daily life is permanently disrupted and mask-wearing is imposed by circumstances.

“*It's going to be become a habit whenever we get sick, like in Asian countries, it's here to stay*”—Woman, mask-wearing, single, age 25, teacher.

### A decision-making process for mask-wearing driven by social relations, common sense, and vulnerability

Interviewees explained that they wear masks in city centers where they feel they are exposed to strangers. Some of them also stopped visiting cities or city centers to not have to wear masks.

“*I wear a mask all the time, it's systematic, when I'm in the street, in shops… Even in the hall of my building when I get home*”—Woman, age 24, postal worker.

Conversely, some participants decided to remove their mask (or to wear it on their hand or at elbow level) in small towns and more generally when they had the feeling of being “in fresh air.”

“*I don't wear one when I'm outside, but in the center, I do, when there are loads of people and we can't necessarily avoid them*”—Man, in a relationship, with children, age 40.

In addition, fear of the police, who checked the mask mandate in large cities, was opposed to the complicity felt among small towns inhabitants, where participants felt they were in a familiar environment, protected because they are with their neighbors and family members. While people wear masks in public transport, this was not the case in their car regardless of the presence of passengers. At home, masks were mainly stored in their entrance halls, at the interface between the security of home and the more uncertain public space.

Most participants declared they did not comply strictly with mask wearing in their private sphere when gathering for drinks or dinners. In return, most ensured that they applied “barrier precautions” and indicated they “kept their distance.” However, during probes, few were able to describe precisely which measures they applied to control transmission. This inability in most respondents to describe practical situations shows that in most cases, physical distancing is more a matter of discourse than a practice.

“*When we go to friends' houses, we don't wear masks but at the same time, we don't get too close to each other and we don't hug*”—Man, in a relationship, age 64, retired.

“*I don't wear one when I have visitors. We tend to believe that the risks are lower, plus we also know that we'll end up having drinks together and will have to take it off then anyway*”—Woman, mask-wearer, single, age 23, doing civic service.

The decision-making for mask wearing relied on what the respondents themselves qualified as “common sense,” based on personal experiential knowledge. This “common sense” was also mentioned as the way of judging the real spread of the epidemic or the real dangerousness of the virus.

“*I'm not a scientist, I trained as a pastry chef originally, but I've studied how bacteria proliferate and I think that wearing masks is more like a culture medium than a barrier. You just need to think a little bit logically to know that it's all crap. Maybe I'm wrong and maybe it is a really serious epidemic and everything… But I go out every day and I see the same people every day and, well, I'm sorry to say this, but no one has disappeared in a year*”—Man, single, age 32, unemployed.

Frequenting people qualified as “vulnerable,” “at risk,” or “fragile” was the only argument that transformed practices. The most repeated public health message that came out in the interviews was: “protect yourself and protect others.” The “others” referred to vulnerable relatives, mainly regarding their age, such as parents or grandparents.

“*We wore masks at Christmas with my whole family, it was really weird… sitting around the table at my grandparents' house. We'd never done it before, it was really strange, it felt clinical when we talked with each other. And it felt weird doing it with close family at a family dinner. But with grandparents and anyone a little bit vulnerable, obviously we tried to be careful… Otherwise, with friends, no, we didn't wear masks*”—*Woman, single, age 26, student*.

Detailed analysis of practices highlighted a strong generational boundary. Young people presented themselves and were presented by their elders as those who were most likely to transmit the virus. The elders presented themselves and were presented by their juniors as those who were most likely to develop a severe form of the virus.

“*In Lyon, we wanted to go and see our grandchildren, but my son said it would be better for us to be vaccinated before going, so we delayed our trip. We didn't even kiss our grandchildren (even though they'd been tested). We used protective measures*”—Woman, in a relationship, age 65, retired.

People under the age of 30 mentioned masks as one of the elements in a toolbox to maintain socialization. Tests were the means often invoked to maintain a link with grandparents. Adults over the age of 60 visiting their own parents did not report this type of practice.

“*For parties or birthdays, we got tested. For Juliette's 18th birthday, last time, the test was obligatory, PCR or antigen […] because it was when there were people from other high schools […]. We got tested 2 days before the party and took care to not see other people in between. And then, during the party, we didn't wear masks anymore. We also tried to not mix glasses and plates, we wrote our names on them*”—Woman, living with her family, age 17, high school student.

### Role of institutions in facilitating incorporation of masks into daily life

Institutions (school and work) played a central role in incorporating appropriate practices regarding the uses and health dimension of masks (Table 3, Q36–40). The people interviewed testified that their employer provided the reason to wear masks and the instructions on how to wear them properly. Then, institutions played a role in transforming mask-wearing into a habit in daily life. While in a private context, people could decide whether or not to wear masks, in the collective context of work or education, they were obliged to adopt the same behavior on a regular, stable, and sustainable basis.

“*At work, we had rubbish bins for biological waste: masks and wipes. We had a protocol when we took a case or returned it*”—Woman, single with children, age 41, salesperson.

The school also played a central role in disseminating good behavior, with teenagers declaring they were very respectful of the rules: regularly changing masks, respecting rules for wearing them.

“*When we went back to school, they taught us how to wear our masks properly […] while before we wore them any old way. We had one for mornings and one for afternoons, and we had to change them at lunchtime*”—Man, living with his family, age 15, high school student.

The practices and habits adopted at work influenced behaviors within the private sphere. Respondents not obliged to wear masks within institutions, did not perceive the need to learn how to wear a mask. Even more, they were convinced that wearing a mask was not something that needed to be learned.

“*Learn how to wear them, no? But after that, I have no idea if I wore them correctly or not*”—Woman, in a relationship, age 45, farmer.

Mimicry seemed to play an important role. In particular, we were able to observe the strength of this socialization with the mask. For example, because the people interviewed wore masks at work, they declared that they also wore masks when they left their home in the morning and all the journey home in the evening.

## Discussion

Using walking interviews with residents from a French region, conducted between April and December 2021 in 11 different cities, this study captured social representations of face masks in the general population. As previously described, masks symbolized the pandemic and transformed daily life (14). Despite proven efficacy, masks were not perceived as a medical device, but as an external constraint. While face masks allowed people to gain some freedom by being protected, overall they remained obstacles to “normal” life, such as that from before the pandemic (15).

The current body of the literature shows that complex social dynamics can emerge from simple individual interactions, and sociocultural variables and local policies can interfere with mask adoption (16). A level of collectivistic thinking in different US states was found to be a robust predictor for a high uptake of face mask use. Where individualistic attitudes were dominant, mask use was lower (17). Social identity, together with in-group favoritism, may be a primary reason for people deciding to be for or against mask wearing in the US, depending on the attitude of their social connections (18). In Spain, the frequency of mask wearing increased when fear increased as a result of disease-related distress or the fear of infecting a close relative or friend who had pre-existing health issues (19). In a previous study, political disagreement appeared to be a factor affecting individual behavior regarding their response to the COVID-19 pandemic (20). Face masks were poorly politicized in our study population. Conspiratorial opinions were an extreme minority. Most of the protest speeches focused essentially on highlighting procrastination in government measures and the confusion generated around the credit to be given to health measures.

The present study is original in that it involves interviews of individuals in the street to obtain their spontaneous expression of the representations and practices of mask-wearing in the specific context of a mask mandate. The use of masks partly revolved around social distancing with relatives. The more individuals belonged to the relative register, the less mask-wearing was felt to be legitimate and justified. The mask was presented by interviewees as a constraint that prevented social interaction. This finding has been described in previous studies, for example by preventing the interpretation of facial expressions (21, 22). People developed avoidance strategies as a reaction to these barriers. In public spaces, masks were worn under the chin, on the hand or elbow, or a certain number of pretext activities (i.e., eating, drinking, or smoking) were used so as not to have to wear masks (8). In private spaces during social gatherings, people said they did not wear masks but applied physical distancing to mitigate risks. However, this notion appeared confusing during interviews. Professional or private gatherings were associated with an increased risk of COVID-19 (19). Spreading events often occurred in poorly ventilated indoor areas with an overall increased risk of transmission. These gatherings in the private sphere need to be targeted for public heath communication and awareness during epidemics.

The decision-making process on how and when to wear masks was based on personal experience and “common sense.” Most people interviewed had never been trained to wear a mask, and more generally had never been educated in preventing transmission. However, they did not feel the need to obtain instructions or advice on this matter. In previous studies, 70–75% of individuals in the public area or during mass gatherings wore their masks correctly (8, 23). This estimate of mask wearing was lower than the 80% threshold suggested as necessary to reduce the spread of COVID-19 (24). This finding has implications for public health. Policies and communication on respiratory protection should not only recommend that masks be used but should also underscore the correct way of using them. Massive education and training efforts need to be provided for the general population on mask wearing to improve protection, as the pandemic is becoming the new normal. In this way, institutions such as work and school seem to be the ideal allies for rendering mask wearing common practice. They created socialization spaces that were favorable for internalizing the health and safety instructions for preventing COVID-19. This context improved understanding of mask wearing from a medical perspective.

### Limitations

The main limitation of this study was that it was carried out in a single French region, with reliance on self-reporting. There may be some factors specific to this setting that influence to what extent our findings can be generalized. No direct observations were performed on mask-wearing. The findings on practices derived from the participants' declarations. Moreover, an observational study was carried out from June to July 2020 to evaluate frequency and quality of facemask use in general populations in the same setting (8). The study was conducted from April to December 2021 in western France. Evolution in the COVID-19 situation and the cultural aspects may influence individual perceptions and representations, with a potential shift over time. Finally, participants were sampled at random. Interviews in public spaces make it possible to obtain information from individuals in the context of mandatory or recommended mask wearing, and where the person interviewed was not likely to have changed their behavior for the sake of social conformity in their relationship with the interviewers.

## Conclusions

This qualitative study emphasizes the need for reinforcement in the individual health and safety value of face masks in the prevention of COVID-19. Ambitious education and training programmes need to be implemented or planned to learn how and when to wear a mask. Institutions (i.e., work and school) may play a critical role in this aspect, providing better and sustainable incorporation of mask-wearing in the general population. From a long-term perspective, system changes should be considered to anticipate the emergence of new respiratory diseases requiring extended use of face masks.

## Data availability statement

The raw data supporting the conclusions of this article will be made available by the authors, without undue reservation.

## Ethics statement

Ethical review and approval was not required for the study on human participants in accordance with the local legislation and institutional requirements. Written informed consent for participation was not required for this study in accordance with the national legislation and the institutional requirements.

## Author contributions

EB and FL participated in the data collection and analysis. GB participated in the data analysis. All authors contributed to the study design and writing. All authors contributed to the article and approved the submitted version.
