# Supplementary figures and images for: Social representations of mask wearing in the general population during the COVID-19 pandemic
Source: Front Public Health. 2023 Apr 24;11:1136980. doi: 10.3389/fpubh.2023.1136980 (PMC10165064; doi:10.3389/fpubh.2023.1136980)

## Appendix

### eFigure

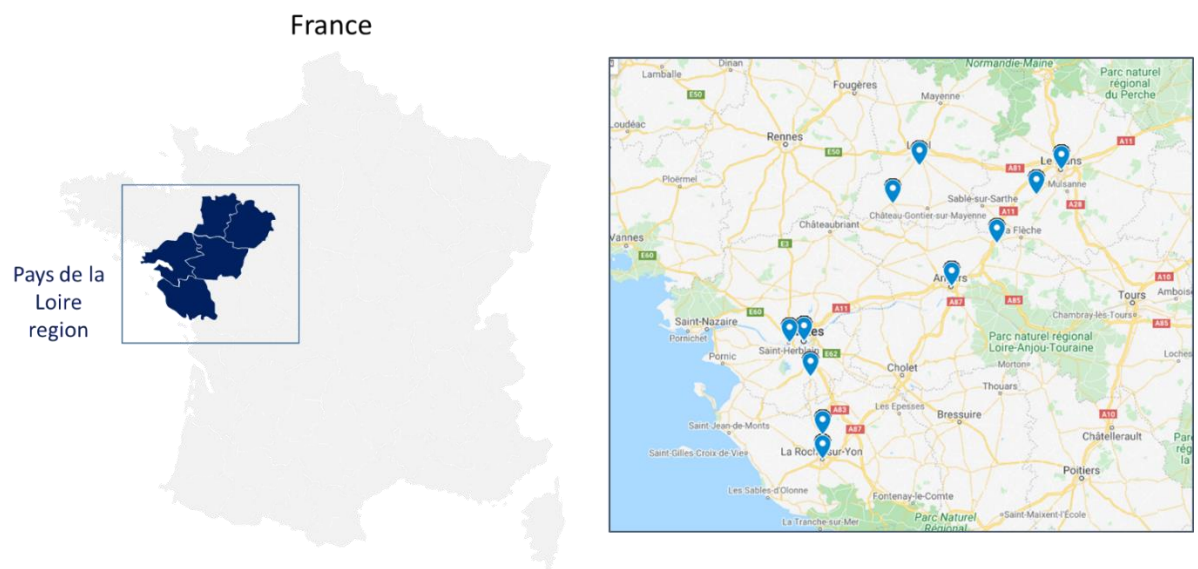

Supplement: Supplementary file 1 [file Image_1.pdf]
